# ChatGPT fails challenging the recent ESCMID brain abscess guideline

**DOI:** 10.1007/s00415-023-12168-1

**Published:** 2024-01-27

**Authors:** Susanne Dyckhoff-Shen, Uwe Koedel, Matthijs C. Brouwer, Jacob Bodilsen, Matthias Klein

**Affiliations:** 1 Department of Neurology with Friedrich-Baur-Institute, LMU University Hospital, LMU Munich (en.), Klinikum Grosshadern of the Ludwig Maximilians University of Munich, Marchioninistr. 15, 81377 Munich, Germany; 2 Department of Neurology, Amsterdam UMC, University of Amsterdam, Amsterdam Neuroscience, Amsterdam, The Netherlands; 3 Department of Infectious Diseases, Aalborg University Hospital, Aalborg, Denmark; 4 Emergency Department, LMU University Hospital, LMU Munich (en.), Munich, Germany; 5 European Society for Clinical Microbiology and Infectious Diseases (ESCMID) Study Group for Infections of the Brain (ESGIB), Basel, Switzerland

**Keywords:** AI, ChatGPT, Brain abscess, Guideline

## Abstract

**Background:**

With artificial intelligence (AI) on the rise, it remains unclear if AI is able to professionally evaluate medical research and give scientifically valid recommendations.

**Aim:**

This study aimed to assess the accuracy of ChatGPT’s responses to ten key questions on brain abscess diagnostics and treatment in comparison to the guideline recently published by the European Society for Clinical Microbiology and Infectious Diseases (ESCMID).

**Methods:**

All ten PECO (Population, Exposure, Comparator, Outcome) questions which had been developed during the guideline process were presented directly to ChatGPT. Next, ChatGPT was additionally fed with data from studies selected for each PECO question by the ESCMID committee. AI’s responses were subsequently compared with the recommendations of the ESCMID guideline.

**Results:**

For 17 out of 20 challenges, ChatGPT was able to give recommendations on the management of patients with brain abscess, including grade of evidence and strength of recommendation. Without data prompting, 70% of questions were answered very similar to the guideline recommendation. In the answers that differed from the guideline recommendations, no patient hazard was present. Data input slightly improved the clarity of ChatGPT’s recommendations, but, however, led to less correct answers including two recommendations that directly contradicted the guideline, being associated with the possibility of a hazard to the patient.

**Conclusion:**

ChatGPT seems to be able to rapidly gather information on brain abscesses and give recommendations on key questions about their management in most cases. Nevertheless, single responses could possibly harm the patients. Thus, the expertise of an expert committee remains inevitable.

## Introduction

Brain abscesses represent a critical and potentially life-threatening central nervous system (CNS) infection [1]. They pose significant diagnostic and therapeutic challenges, often requiring urgent medical intervention to prevent severe neurological complications or even death [2]. Historically, the management of brain abscesses has largely been guided by clinical experience and only limited studies; furthermore, an international guideline has been non-existing until recently [1]. Recognizing this gap, the European Society of Clinical Microbiology and Infectious Diseases (ESCMID) Study Group for Infections of the Brain (ESGIB) took the initiative to develop a structured clinical guideline, addressing this need for a standardized approach [3]. Central to this guideline’s creation was the formulation and assessment of ten PECO (Population, Exposure, Comparator, Outcome) questions, intended to cover the most pertinent and debated aspects of brain abscess management [3].

At the same time, the rise of artificial intelligence (AI) has heralded a transformative era in various scientific domains, including medicine [4]. The ability of AI to rapidly assimilate, process, and interpret vast data sets offers a tantalizing prospect: Are time-consuming processes to create guidelines still necessary or could AI models, trained on a wealth of medical literature, rival clinical experts in answering complex clinical questions? With AI poised to become a fundamental part in clinical research and decision-making, this study sought to evaluate its potential by pitting ChatGPT, a state-of-the-art AI language model, against the newly minted ESCMID guideline on management of brain abscesses. Specifically, we were interested in discerning whether ChatGPT could competently answer the same ten PECO questions that were central to the guideline’s formation, thereby providing insights into AI’s capability to support evidence-based clinical decision-making. While other studies have already tried to pit ChatGPT against medical guidelines, our study is the first not only in the neurological field but also to directly compare the recommendations of the AI program with medical experts by feeding the same scientific literature into the algorithm that was used for the guideline development.

## Methods

The primary aim of this study was to assess the concordance between ChatGPT’s recommendations on brain abscess diagnostics and treatment, derived from two different approaches, with the recommendations of the ESCMID guideline.

The study utilized ten key questions that were initially developed and appraised by the ESCMID committee for their brain abscess guideline including areas of diagnostic strategies and therapeutic modalities pertinent to brain abscesses.

The first approach involved direct querying of ChatGPT. For each key question, a new chat was used. Each of the ten questions was posed directly to ChatGPT (version 4.0) without any additional context or information. To achieve greater comparability between responses, ChatGPT was then prompted to answer the key question in two sentences. ChatGPT’s responses were documented verbatim for subsequent comparison. Next, ChatGPT was questioned on the grade of evidence and the strength of its recommendation, each in one sentence.

The second approach represented informed Querying of ChatGPT: before posing the same ten questions to ChatGPT (version 4.0), the AI was primed with data extracted from the studies that the ESCMID committee used in formulating their guideline (literature was identified through a structured literature review process [3]). This priming involved presenting the text from these studies to the AI model. Once primed, the same questions were asked, and responses were again documented verbatim.

The responses obtained from both the direct and informed approaches were independently compared against the recommendations from the ESCMID guideline. This comparison was carried out by three independent reviewers with expertise in infectious CNS diseases (MB, JB, MK) who—as ESGIB members—also played a leading role in the development of the ESCMID guideline.

For a comprehensive evaluation of AI’s recommendations on questions about brain abscess, three scores were obtained (Table 1):

**Table 1:** Scores evaluating the response quality of ChatGPT

Clarity of the recommendation: Does ChatGPT give a clear response?		Alignment Score (adapted according to Cakir et al. [5])		Patient risk score	
(a)	Yes, concrete	1 point	Completely correct (ChatGPT's recommendation matches the ESCMID guideline completely)	(a)	Recommendation presents no patient hazard
(b)	Yes, but incomplete	2 points	Correct but inadequate (ChatGPT's recommendation somewhat matches but lacks the full depth/detail of the ESCMID guideline)	(b)	A patient hazard cannot be ruled out
(c)	No	3 points	A mix of correct and misleading information (ChatGPT's recommendation diverges significantly but may have some minor overlap with the ESCMID guideline)	(c)	The recommendation poses a high risk of patient harm
		4 points	Completely incorrect (ChatGPT's recommendations directly contradicts the ESCMID guideline)

(i) The first criterion reflected the clarity of the AI model’s recommendation: (a) if a concrete recommendation was provided, (b) if a recommendation was given, but incomplete, and (c) if no clear recommendation was provided.
(ii) Next, we employed an Alignment score, that was adapted from Cakir et al. [5] to match our study design: 1 point: completely correct match with the ESCMID guideline, 2 points: correct, but inadequate (some overlap, but lacking the complete depth of the ESCMID guideline), 3 points: a mix of correct and misleading information (significant divergence from guideline with minor overlap) and 4 points: completely incorrect (direct contradiction to the ESCMID guideline). A mean score ≤ 2.0 was rated as correct, while a mean score > 3.3 was evaluated as completely incorrect. Scores > 2.0 and ≤ 3.3 indicated mixed answers with correct and incorrect parts.
(iii) The last assessment concerned the risk of patient harm due to ChatGPT’s recommendation: (a) recommendation presents no patient hazard, (b) a patient hazard cannot be ruled out, (c) high risk of patient harm.

Scores attributed to the recommendations by the three reviewers were averaged for each response, providing a consensus score for each of the two approaches per question. The scores for response clarity, alignment and patient risk were analyzed for each approach thus indicating the quality of ChatGPT’s recommendations. Fleiss kappa values for interrater reliability were calculated using SPSS (version 29).

## Ethics approval

As there were no human participants used in this study, an ethics board approval is not applicable.

## Results

### ChatGPT provided mostly clear responses to key questions on brain abscess

The clarity of ChatGPT’s recommendations were valued between a (yes, concrete) and b (yes, but incomplete) in 80% of responses (Table 2). When answering key questions #2 on withholding antimicrobials until neurosurgery and #10 on primary-prophylactic antiepileptic treatment, the AI’s responses were deemed not clear enough to guide physicians in respect to the question asked. In the survey with data prompting, clarity of answers was overall slightly better (90%), including answers for key questions #2 and #10.

**Table 2:** 10 PECO questions on brain abscess with recommendations, grade of evidence and strength of recommendation by ChatGPT without and after data entry, compared to the ESCMID guideline. In another column, the score assessments of 3 reviewers on clarity, alignment with the ESCMID guideline and patient hazard are added. Fleiss kappa values at the bottom of the table indicate interrater reliability

	ChatGPT (without data)						ChatGPT (with data)						ESCMID guideline		
Key question	Recommendation	Grade of evidence	Strength of recommen-dation	Clarity of recommen-dation	Alignment with ESCMID	Patient risk score	Recommendation	Grade of evidence	Strength of recommen-dation	Clarity of recommen-dation	Alignment with ESCMID	Patient risk score	Recommendation	Grade of evidence	Strength of recommendation
1: What is the preferred brain imaging modality in patients with suspected brain abscess?	Magnetic resonance imaging (MRI) with contrast is the preferred brain imaging modality for patients with suspected brain abscess because it offers superior soft-tissue resolution and can better differentiate abscesses from other intracranial lesions. Computed tomography (CT) with contrast can also detect brain abscesses, but MRI is more sensitive and specific for this purpose.	Low	Strong	b, a, a	2.0 (2, 2, 2)	a, a, a	Based on the information provided in this chat, the preferred brain imaging modality for patients with suspected brain abscess is CE-SWI (Contrast Enhanced Susceptibility Weighted Imaging). However, it's crucial to examine the original magnitude and phase images carefully due to potential post-processing artifacts.	Observa-tional	Moderate	b, b, c	3.3 (3, 3, 4)	a, a, a	We strongly recommend brain MRI including DWI/ADC and T1 weighted imaging with and without gadolinium for patients with suspected brain abscess. If MRI is not available, contrast-enhanced CT is recommended.	High	Strong
2: Should antimicrobials be withheld until aspiration or excision in patients with suspected brain abscess?	No, in patients with suspected brain abscess, empiric antimicrobial therapy should be initiated promptly after obtaining necessary cultures (preferably before administering antibiotics, if possible). Delaying antimicrobial treatment can lead to disease progression and worsened outcomes, while obtaining a sample for culture prior to antibiotic initiation ensures the best chance of identifying the causative organism.	Moderate to low	Strong	b, b, c	3.3 (4, 3, 3)	b, a, a	Based on the information provided in the chat, most studies suggest that withholding antimicrobials until aspiration or excision in patients with suspected brain abscess might lead to better culture yields. Therefore, it is recommended to consider withholding antimicrobials in such cases to optimize diagnostic accuracy, unless clinically contraindicated.	Moderate to strong	Strong	a, b, c	1.3 (1, 1, 2)	a, NA, a	We conditionally recommend that antimicrobials are withheld until aspiration or excision of brain abscess in patients without severe disease if neurosurgery can be carried out within reasonable time, preferably within 24 hours of radiological diagnosis.	Low	Conditional
3: Should molecular-based diagnostics be used in patients with brain abscess?	Molecular-based diagnostics, such as polymerase chain reaction (PCR) and next-generation sequencing, can be useful in identifying causative organisms in brain abscesses, especially when conventional cultures are negative or when atypical pathogens are suspected. Their use can supplement traditional culture methods and provide more comprehensive pathogen identification, particularly in challenging cases.	Moderate to low	Moderate	b, b, a	1.3 (1, 2, 1)	a, a, a	Molecular-based diagnostics offer rapid and accurate identification of pathogens in patients with brain abscesses and can be especially beneficial when traditional culture methods are inconclusive. However, it's essential to interpret results alongside clinical findings and ensure proper sample collection to avoid contamination.	*-*	*-*	b, a, a	1.7 (3, 1, 1)	a, NA, a	We conditionally recommend the use of molecular-based diagnostics, if available, in patients with negative cultures.	Moderate	Conditional
4: Should neurosurgical aspiration or excision be used in patients with brain abscess?	Neurosurgical aspiration or excision is indicated in patients with brain abscesses for both diagnostic and therapeutic purposes. Factors favoring surgical intervention include larger abscess size (usually >2.5 cm), location (e.g., in the cerebellum where mass effect is poorly tolerated), failure of medical therapy, or the need for definitive microbiological diagnosis when cultures are negative or unclear.	Moderate to low	Strong	b, b, a	2.0 (3, 2, 1)	b, a, a	Based on the information provided in this chat, early surgical intervention, either through neurosurgical aspiration or excision, is associated with a reduced risk of treatment failure in patients with brain abscesses, especially in specific conditions. Therefore, neurosurgical intervention is recommended for patients with brain abscesses, considering individual clinical presentations and circumstances.	Moderate	Moderate	b, a, a	1.7 (2, 2, 1)	a, a, a	We strongly recommend neurosurgical aspiration or excision of brain abscess as soon as possible whenever feasible (excl. toxoplasmosis).	Moderate	Strong
5: What is the optimal empirical antimicrobial therapy for brain abscess?	The optimal empirical antimicrobial therapy for brain abscess typically targets a combination of aerobic and anaerobic bacteria, including streptococci, staphylococci, and gram-negative bacilli. Commonly used regimens include a third or fourth-generation cephalosporin (e.g., ceftriaxone or cefotaxime) combined with metronidazole; if there's concern for MRSA, vancomycin or linezolid can be added.	Moderate to low	Strong	b, a, a	2.0 (2, 2, 2)	a, a, a	Based on the provided studies in this chat, the optimal empirical antimicrobial therapy for brain abscess includes a combination of third-generation cephalosporins (such as cefotaxime or ceftriaxone), metronidazole, and vancomycin. This regimen covers a broad spectrum of the commonly implicated pathogens in brain abscesses.	Low	Moderate	a, a, a	2.0 (2, 2, 2)	a, a, a	We strongly recommend 3^rd^ generation cephalosporin combined with metronidazole for empirical treatment of community-acquired brain abscess in children and adults (Suppl. Table 3). Dosages should be tailored toward adequate abscess and central nervous system penetration (Suppl. Table 10).	Low	Strong
6: What is the appropriate duration of antimicrobial therapy for bacterial brain abscess?	The appropriate duration of antimicrobial therapy for bacterial brain abscess typically ranges from 6 to 8 weeks. The exact duration may vary based on the causative organism, the clinical response to treatment, and whether surgical intervention was undertaken, with some cases requiring a longer duration of therapy based on individual circumstances.	Moderate to low	Strong	b, a, a	1.7 (2, 2, 1)	a, a, a	The appropriate duration of antimicrobial therapy for bacterial brain abscess, based on the provided information, is intravenous administration for about 4 weeks, followed by 12 weeks of oral medication. This recommendation is derived from the study's strategy, which achieved high cure rates with minimal treatment-related adverse events.	–	–	b, a, a	3.7 (4, 4, 3)	b, b, a	We conditionally recommend a total duration of 6-8 weeks of intravenous antimicrobials for aspirated or conservatively treated brain abscesses. Based on expert opinion, a shorter duration of four weeks may be considered in patients treated with excision of brain abscess.	Low	Conditional
7: Should early transition to oral antimicrobials be used in treatment of patients with bacterial brain abscess?	Early transition to oral antimicrobials in the treatment of bacterial brain abscess can be considered in select patients who have had a good clinical response to intravenous therapy and when a suitable oral agent with good CNS penetration is available. However, the decision should be individualized based on the causative organism, abscess size, clinical progress, and availability of oral agents with adequate bioavailability and brain penetration.	Moderate to low	Moderate	b, b, b	2.0 (2, 2, NA)	a, a, a	Based on the information provided, an early switch to oral antibiotics during the first 14 days of treatment for brain abscesses seems to be associated with favorable outcomes in selected patients. However, due to the limitations of the studies, including their retrospective nature, it's recommended to proceed with caution, and more robust, prospective research is needed to confirm these findings.	Moderate	Conditional	b, b, b	4.0 (4, 4, NA)	b, b, a	No recommendation. For early transition to oral antimicrobials in patients with brain abscess, there is insufficient evidence at the time of writing to provide a recommendation.	-	-
8: Should consolidation therapy with oral antimicrobials after ≥6 weeks of IV antimicrobials be used to reduce risks of relapse or recurrence?	Consolidation therapy with oral antimicrobials after ≥6 weeks of IV antimicrobials for bacterial brain abscess can be considered, especially in cases where there are concerns about incomplete resolution or potential for relapse. However, the need and duration for such consolidation therapy should be individualized based on clinical and radiological progress, the causative organism, and the risk factors associated with the patient.	Moderate to low	Moderate	b, b, a	2.3 (3, 2, 2)	a, a, a	Based on the provided data, the study does not explicitly mention or provide evidence regarding the efficacy of consolidation therapy with oral antimicrobials after ≥6 weeks of IV antimicrobials for the prevention of relapse or recurrence in pediatric brain abscess patients. Therefore, a clear recommendation cannot be made based on the given information.	Very low	Conditional	a, c, a	3.0 (3, 3, 3)	a, a, a	Based on expert opinion, we conditionally do not recommend oral consolidation treatment after ≥6 weeks of IV antimicrobials for brain abscess (excl. permanent neuroanatomical defects, tuberculosis, nocardiosis, toxoplasmosis, and fungal brain abscess).	Very low	Conditional
9: Should dexamethasone be used to treat severe symptoms due to perifocal oedema in patients with brain abscess?	Yes, dexamethasone can be used to treat severe symptoms due to perifocal edema in patients with brain abscess. It can help reduce cerebral edema and alleviate increased intracranial pressure, but its use should be short-term and typically tapered as symptoms improve to minimize potential side effects.	Moderate to low	Strong	a, a, a	1.3 (1, 2, 1)	a*, a, a	Based on the provided data, dexamethasone shows a potential benefit in reducing the mortality and surgical intervention rate in patients with brain abscess experiencing severe symptoms due to perifocal oedema. However, given the observational nature of the data, the recommendation should be approached with caution, and further randomized controlled trials are needed to confirm its efficacy.	Low	Weak	b, b, b	2.7 (3, 3, 2)	a, a, a	In the absence of convincing clinical data of harm related to adjunctive corticosteroid treatment, we strongly recommend use of corticosteroids for management of severe symptoms due to perifocal oedema or impending hernation in patients with brain abscess.	Low	Strong
10: Should primary prophylaxis with antiepileptic treatment be used to reduce risks of seizures during admission and subsequent epilepsy?	As of my last update in September 2021, primary prophylaxis with antiepileptic drugs (AEDs) for patients with brain abscess to reduce the risk of seizures during admission and prevent subsequent epilepsy remains controversial. Some guidelines recommend short-term prophylaxis during the acute phase, especially if there are risk factors for seizures, while others suggest it may not be necessary for all patients and emphasize the potential side effects and drug interactions associated with AEDs.	Moderate to low	Moderate	c, c, c	3.0 (3, 3, 3)	a, a, a	Based on the information provided, the text does not directly address the use of primary prophylaxis with antiepileptic treatment for patients with brain abscesses. Therefore, given the available data, it is not possible to make a definitive recommendation on the use of antiepileptics for primary prophylaxis in this patient population.	–	–	a, a, a	2.7 (2, 2, 4)	a, a, a	Based on expert opinion, we conditionally recommend against primary prophylaxis with antiepileptics in patients with brain abscess.	Very low	Conditional
Fleiss Kappa				0.362	0.419	0.548				0.389	0.453	0.660			

### Without data prompting, ChatGPT gave more correct recommendations than with data input

Regarding the alignment of ChatGPT’s responses with the ESCMID guideline, a score from 1 (completely correct) to 4 points (completely incorrect) was raised. Overall, the mean score without data input (2.1 points) was significantly better than with data input (2.6 points). Without data prompting, the AI answered 70% of the key questions correctly (score ≤ 2.0). ChatGPT gave recommendations on withholding of antimicrobials (#2), consolidation therapy (#8) and primary prophylaxis with antiepileptics (#10) not aligning with the ESCMID guideline. No recommendation by ChatGPT directly contradicted the ESCMID guideline. In the second survey after data entry, only 40% of key questions were answered correctly (score ≤ 2.0). In 60% of questions, alignment with the ESCMID guideline was lower after data entry than without data entry. Moreover, responses on the appropriate duration of antimicrobial therapy (#6) and on early transition to oral antimicrobials (#7) even contradicted the ESCMID guideline directly after data entry and were considered completely incorrect (score > 3.3).

Fleiss kappa values for interrater reliability in the assessment of the alignment score were 0.419 (without data entry) and 0.453 (with data entry) indicating moderate agreement (Table 2).

### Patient hazard was possible in two recommendations by ChatGPT

At last, ChatGPT’s recommendations were analyzed on their potential to constitute a patient hazard. Overall, almost all recommendations by ChatGPT were estimated of presenting no patient hazard. Interestingly, one reviewer assessed ChatGPT’s answer without data prompting on the use of dexamethasone in brain abscess (#9) as even better than the ESCMID guideline’s recommendation. However, for the AI’s responses on key questions #6 and #7 after data input, which directly contradicted the ESCMID guideline, two out of three experts judged that a patient hazard cannot be ruled out if ChatGPT’s recommendation were followed.

### ChatGPT provided the grade of evidence and strength of its recommendations

When asked, ChatGPT provided estimations on the grade of evidence and strength of recommendation for most of its recommendations. In longer versions of ChatGPT’s answers (data not shown), the AI model repeatedly used the GRADE (Grading of Recommendations, Assessment, Development and Evaluation) system [6] to evaluate the strength of its recommendation and grade of evidence. Without data input, grade of evidence was rated in six out of nine questions similar to the ESCMID rating, the strength of recommendation in seven out of nine questions. The ESCMID guideline did not provide a rating for key question #7. After data input, ChatGPT only provided the grade of evidence and strength of recommendation for seven of its recommendations. The grade of evidence was similar to the ESCMID rating in four out of six questions, but the strength of recommendation only in one out of six recommendations. In both surveys, alignment of grade of evidence and strength of recommendation with the ESCMID rating was not associated with the alignment of the content of recommendation.

### Additional remarks

As the study was conducted before the publication of the new ESCMID guideline on brain abscesses, ChatGPT stated frequently that it was working with data up until September 2021 and did not have access to any more current data. Moreover, at the end of each recommendation (in the longer versions, data not shown), the AI model stated that these decisions in patients with brain abscesses should be made in consultation with a multidisciplinary team, including infectious disease specialists, neurologists, and neurosurgeons. It also added that as medical knowledge and practices evolve, the most current guidelines should be consulted.

## Discussion

In summary, ChatGPT was able to give recommendations on the management of patients with brain abscess for most of the key questions, including assessment of grade of evidence and strength of recommendation. Without data prompting, 70% of questions were answered correctly and no patient hazard was present. However, in 30% of the cases, it did not come up with a correct or nearly correct advice. Although data input slightly improved the clarity of ChatGPT’s recommendations, it led to less correctly answered key questions and two recommendations were found to be directly contradicting the guideline. Alarming is the fact, that a patient hazard seemed possible if ChatGPT´s advice was followed.

The AI’s knowledge was from before September 2021 and it had no access to more current data such as the new ESCMID guideline from 2023. It must be added that the key questions in this study cover extremely complex medical issues, some of which are controversial even among experts and for some of which hardly any robust data are available, which was one of the reasons for drawing up the guideline. As we knew which studies had been included in the ESCMID committee’s answers to the key questions, we tried to optimize ChatGPTs outcome by entering the studies into ChatGPT, assuming that it would result in answers closer to the ESCMID guideline. However, our results showed impressively that this was not the case, but that, on the contrary, the recommendations after data entry align less with the ESCMID guideline. For the first approach, ChatGPT presumably drew on a wider pool of literature, including non-scientific literature. Yet for the second approach, the same scientific studies that were screened, reviewed, and evaluated for the guideline development following a strict protocol, were fed into the AI algorithm. The fact that ChatGPT’s recommendations were inferior after data entry—especially in two PECOs—might be due to an overvaluation of the few observational studies provided for key questions 6 and 7, for one of which even the guideline panel was not able to give a recommendation as the evidence was rated insufficient to answer the question. As the exact operating procedures of ChatGPT remain intransparent, we hypothesized that while the AI model is able to process large amounts of data quickly, it may lack the ability to correctly classify and weight the data based on their scientific quality. Moreover, ChatGPT only seemed to take the last chat entry into consideration for answering the key question (#1), leading to a wrong response. It remains unclear which data are exactly used for ChatGPT’s responses as the exact proceedings of the AI could not be traced. It can, therefore, be concluded that data entry of studies into ChatGPT is not necessarily improving medical recommendations. It should be noted though, that our findings are a temporary observation and a re-evaluation of the recommendation quality of ChatGPT should be reviewed on an ongoing basis following its evolution and further development.

Of note, kappa values for interrater reliability showed only moderate agreement in the assessment of alignment between ChatGPT and the guideline. The three reviewers being part of creating the ESCMID guideline might have influenced their assessments of concordance of recommendation. To mitigate this risk, predefined scores were used to render the assessments more objectifiable.

ChatGPT has already been tested and compared to several clinical guidelines from varying medical departments, for example for treatment of advanced solid tumors [7], spine surgery [8, 9], urology [10], and diabetic ketoacidosis [11, 12].

On the topic of post-colonoscopy management, ChatGPT provided responses with 90% adherence to guidelines and 85% accuracy [13], suggesting beneficial use for healthcare providers and patients. ChatGPT’s recommendations on the management of lumbar spinal stenosis were also in line with findings in the current literature [9]. When asked guideline-based questions on urological topics, ChatGPT provided only 60% appropriate responses [10]. The authors of the study criticize misinterpretation of clinical care guidelines as well as dismissal of important context by the AI. Similarly, the agreement between answers by ChatGPT and guideline recommendations on five hepato-pancreatico-biliary conditions lay at 60% as well [14].

In this context, the accuracy rate of ChatGPT in our study appears to be in the range of previous comparisons of AI’s recommendations with medical guidelines. Since there was no assessment of ChatGPT’s recommendations on another neurological disease before, our findings add value to the previous results as the medical knowledge of the AI program should be assessed on a broad spectrum of diseases and medical departments.

Inconsistencies in the repeated reportings did not only occur in our survey with ChatGPT, but was also observed in other studies on the efficacy and reproducibility of ChatGPT [11, 15].

The most important limitation of current AI models lies in the lack of transparency: the fact that ChatGPT does not disclose the sources of its answers, consequently results in a risk of dubious literature being used that the user neither track, verify or control. We hypothesized that ChatGPT might be more accurate in the interpretation of RCTs than observational studies thus leading to more imprecise answers particularly in the brain abscess field where large RCTs are lacking. It also remains unclear to what extent ChatGPT is able to analyze data and assign them different levels of credibility depending on the risks of bias and confounding and interpreting them. In longer versions of its responses to the key questions (not shown), ChatGPT added that medical experts should be consulted and the most recent knowledge and guidelines should be used for clinical decision-making, thus attenuating its recommendations and acknowledging the fact that blindly relying on AI might put patients at risk.

## Conclusion

While ChatGPT presents a valuable adjunctive tool in broad clinical contexts at first sight, wrong recommendations were given to single questions. This is alarming as it appears too dangerous to trust on recommendations given by ChatGPT in a medical context. The nuanced expertise of specialized committees remains essential, especially for complex clinical queries. As ChatGPT continues to evolve, it is necessary to reevaluate this question in the future.

## Data Availability

Data not provided in the article because of space limitations may be shared at the request of any qualified investigator for purposes of replicating procedures and results.
